# Receptor-Receptor Interactions as a Widespread Phenomenon: Novel Targets for Drug Development?

**DOI:** 10.3389/fendo.2019.00053

**Published:** 2019-02-18

**Authors:** Diego Guidolin, Manuela Marcoli, Cinzia Tortorella, Guido Maura, Luigi F. Agnati

**Affiliations:** ^1^Department of Neuroscience, University of Padova, Padova, Italy; ^2^Department of Pharmacy and Center of Excellence for Biomedical Research, University of Genova, Genoa, Italy; ^3^Department of Biomedical Sciences, University of Modena and Reggio Emilia, Modena, Italy; ^4^Department of Neuroscience, Karolinska Institutet, Stockholm, Sweden

**Keywords:** allosteric modulation, oligomerization, receptors, GPCR, signaling, pharmacology

## Abstract

The discovery of receptor-receptor interactions (RRI) has expanded our understanding of the role that G protein-coupled receptors (GPCRs) play in intercellular communication. The finding that GPCRs can operate as receptor complexes, and not only as monomers, suggests that several different incoming signals could already be integrated at the plasma membrane level via direct allosteric interactions between the protomers that form the complex. Most research in this field has focused on neuronal populations and has led to the identification of a large number of RRI. However, RRI have been seen to occur not only in neurons but also in astrocytes and, outside the central nervous system, in cells of the cardiovascular and endocrine systems and in cancer cells. Furthermore, RRI involving the formation of macromolecular complexes are not limited to GPCRs, being also observed in other families of receptors. Thus, RRI appear as a widespread phenomenon and oligomerization as a common mechanism for receptor function and regulation. The discovery of these macromolecular assemblies may well have a major impact on pharmacology. Indeed, the formation of receptor complexes significantly broadens the spectrum of mechanisms available to receptors for recognition and signaling, which may be implemented through modulation of the binding sites of the adjacent protomers and of their signal transduction features. In this context, the possible appearance of novel allosteric sites in the receptor complex structure may be of particular relevance. Thus, the existence of RRI offers the possibility of new therapeutic approaches, and novel pharmacological strategies for disease treatment have already been proposed. Several challenges, however, remain. These include the accurate characterization of the role that the receptor complexes identified so far play in pathological conditions and the development of ligands specific to given receptor complexes, in order to efficiently exploit the pharmacological properties of these complexes.

## Introduction

The concept of “receptor” was independently proposed by Ehrlich and Langley (1) at the beginning of the 20th century to explain the selective effects of drugs and suggested that the action of a drug involved the formation of specific complexes with molecular agents in the target cells, thereby eliciting a cell response. In the decades that followed, this hypothesis was demonstrated, receptor molecules were biochemically identified, and their structures discovered, thus enabling the key role that they play in physiology to be fully understood. More than 4% of the human genome encodes cell receptors (2); these are organized into different families [see (3)] including matrix receptors (e.g., integrins), ligand-gated (LGIC, 76 members in the human genome) and voltage-gated (VGIC, 143 members) ion channels, intracellular receptors, such as nuclear hormone receptors (NHRs, 48 members), enzyme-linked receptors, such as receptor tyrosine kinases (RTKs, 58 members), and G protein-coupled receptors (GPCRs). GPCRs constitute the largest family; in mammals, they contribute to almost all physiological processes and are currently very common targets for drugs (2, 4). In humans, the GPCR family is made up of about 800 receptors; these are classified in five major groups, namely classes A (the largest group), B, C, frizzled, and adhesion (5), mainly on the basis of their structural and functional similarities (6). GPCRs have a highly conserved overall structure [see (7, 8)], exhibiting seven α-helixes that span the plasma membrane (transmembrane domains, TM) and are connected to one another by extra- and intracellular loops (ECL and ICL). The stability of the TM region is provided by interhelical bonds and hydrophobic interactions between highly conserved residues. The extracellular domain (encompassing the N-terminus of the protein) displays high structural variability among the different classes of GPCRs, being very large in class C GPCRs (9). In several GPCRs (e.g., class C GPCRs) it is the domain that hosts the ligand-binding site, while in others (e.g., most of class A GPCRs) the ligand-binding pocket is positioned in the extracellular half of the TM bundle (10). When ligand binding occurs, it induces a conformational change of the TM core, allowing the activation of downstream signaling pathways. *In vitro* and *in vivo* experiments have demonstrated that GPCRs can recognize and decode signals (of chemical or physical nature) as monomers. On this issue, studies of particular interest have shown that monomers of three class A GPCRs (namely rhodopsin, β_2_-adrenergic, and μ-opioid receptors) trapped inside nanodiscs are able to signal (11–13). In addition, intrinsic plasticity has been found to characterize signaling from GPCR monomers, in that they can assume multiple active conformations because of their binding with ligands, thereby initiating different patterns of signal transduction [see (14)], such as G protein and/or arrestin pathways (15).

However, evidence of negative cooperativity between β-adrenergic receptors has also emerged (16) and in the 1980 s *in vitro* and *in vivo* experiments by Agnati et al. (17, 18) and Fuxe et al. (19) provided indirect biochemical and functional evidence that structural receptor-receptor interactions (RRI) could be established between GPCR monomers [see (20) for further historical details]. These findings led to the hypothesis that supramolecular complexes of receptors consisting of different types of GPCRs could form at the cell membrane and could modulate synaptic weight (21), probably affecting learning and memory processes (22). It was also suggested that receptor–receptor interactions could allow the integration of synaptic (wiring transmission) and extrasynaptic (volume transmission) signals (23), one of the mechanisms underlying the appearance of polymorphic networks [see (24)]. The term RRI was subsequently proposed in order to emphasize the concept of an interaction between receptor proteins that required direct physical contact between the receptors and which led to the formation of dimers or high-order oligomers at the cell membrane. The first observations indicating the dimerization of GPCRs were made by Fraser and Venter (25) and by Paglin and Jamieson (26), and a breakthrough in the field of RRI came with the discovery of the GABA_B_ receptor heterodimer (27). In the years that followed, the existence of receptor complexes formed by GPCRs was supported by more direct evidence provided by several groups, and the amount of available data increased significantly with the development (and widespread diffusion) of biophysical techniques aimed at detecting the spatial proximity of protein molecules [see (8, 28) for reviews].

It is now well recognized that class C GPCRs constitutively form homomers or heteromers (29) and some evidence has also suggested that class B GPCRs could also be involved in oligomerization processes [see (30, 31)]. With regard to class A GPCRs, their involvement in receptor complex formation in living tissues is debated [see (32)]. Indeed, some authors contend that no single experimental approach can, as yet, conclusively demonstrate these complexes *in vivo* (33). The possibility of class A GPCR complexes in native systems, however, is strongly supported by the available evidence as a whole. Indeed, several different approaches have provided consistent results pointing to the existence of class A GPCR complexes (34). Moreover, it should be noted that the above-mentioned class A GPCRs able to signal as monomers have also been seen to form receptor complexes (35–37). Thus, the existence of functional assemblies of class A GPCRs cannot be excluded [a discussion of this topic was recently provided by Franco et al. (38)]. In this respect, interesting studies have shown that a monomer-dimer equilibrium characterizes class A GPCRs in the cell membrane, where the half-lives of dimers (as determined from the rate of association and dissociation) indicate that they are often transient (39). This may help explain opposing views on the role of class A GPCR oligomerization (40).

The number of RRI involving GPCRs that have been identified so far is quite high and continuously increasing [see (7, 8) for recent reviews]. Most of these are stored in the GPCR Oligomerization Knowledge Base [http://www.gpcr-okb.org (41)], and, for what concerns the heteromers, in the GPCR-HetNet [http://www.iiia.csic.es/~ismel/GPCR-Nets/index.html (42)], which together comprise more than 500 entries. The research that has yielded most of these findings has focused on neurons and synapses [see (43)]. RRI between GPCRs, however, have also been seen to occur in other cell types and in districts other than the central nervous system (CNS). Furthermore, direct RRI involving the formation of receptor complexes is a feature observed in the other families of receptor molecules [see (44)]. Thus, RRI appear as a widespread phenomenon, and oligomerization as a common mechanism for receptor function and regulation.

Allosteric interactions [see (45)] are the basic molecular mechanism underlying the formation of these receptor assemblies. As recently outlined by Changeux and Christopoulos (44), the monomers forming these assemblies display a cooperative behavior, which is enabled by the action of orthosteric and allosteric ligands. Thus, the cell-decoding apparatus becomes endowed with elaborate dynamics in terms of recognition and signaling. To emphasize the “integrated output” of this input unit, the term “receptor mosaic” (RM) was also proposed, in order to indicate a multiple assembly of receptors (46). This term, indeed, stressed the concept that the emergent properties of the assembly depend not only on the type of allosteric interactions (entropic and/or enthalpic) within the integrative complex (47, 48), but also on the location and the order of activation of the participating receptors (49). On this basis, the suggestion was made (50–52) that RRI could pave the way to new strategies aimed at new targets for drug treatment. In recent years this idea has become the subject of intense research to identify receptor complexes that could constitute promising targets for the treatment of pathological conditions, and novel pharmacological strategies have already been proposed [see (7, 28, 53) for recent reviews].

Here, we will briefly review the available data on the occurrence of direct RRI between receptor proteins, the fundamentals of receptor complex formation and the impact that receptor oligomerization may have from a pharmacological standpoint.

## RRI as a Widespread Phenomenon

In recent decades, GPCRs have become the main focus of studies aimed at characterizing RRI, with specific regard to the CNS. Indeed, the formation of receptor complexes is considered to be of key importance in neurophysiology (7), especially in the emerging field of “connectomics” [see (43) for a review], since integration of the input signals, already at the level of the plasma membrane, can significantly contribute to setting and tuning synaptic strength and, more generally, the efficiency of intercellular communication. Furthermore, receptor complexes may be of great importance in neuropsychopharmacology [see (7, 28, 53–55) for extensive recent reviews], and have become appealing potential targets for the development of novel therapeutic strategies in serious diseases of the CNS, such as depression and schizophrenia [see (50, 56)], Parkinson's disease [see (57)], addiction (52), neuropathic pain (58), and eating disorders (59).

GPCR homomers and heteromers, however, can be found in cell types other than the central neurons, and receptor oligomerization is not limited to GPCRs.

## GPCR Complexes in Astrocytes

In the CNS, astroglia constitutes the main glial population, and increasing evidence suggests that, at the level of excitatory synapses, neurons and astrocytes interact bidirectionally, a finding that has led to the proposal of the concept of the “tripartite synapse” (60). To monitor the extracellular environment [see (57, 61)] astrocytes express specific receptors and channels, the activation of which elicits Ca^2+^ responses in the cells (62); these responses can, in turn, induce the release of gliotransmitters (glutamate, D-serine, ATP), thereby actively modulating synaptic transmission (63). Specifically, there is evidence that adult striatal astrocytes express both adenosine A_2A_ receptors (64) and D_2_ receptors for dopamine (65). Interestingly, *in vivo* studies have indicated that astrocytic A_2A_ receptor dysfunction disrupts glutamate homeostasis (66), while D_2_ receptors modulate immune responses in neuroinflammation-associated disorders and increase the resistance of neurons to toxic damage (67).

A considerable number of investigations conducted on these GPCRs in cell models have demonstrated that, when D_2_ and A_2A_ receptors are expressed on the same cell, they can interact and heterodimerize (68–70). Moreover, functional and physical evidence has shown that, in striatal neurons, native A_2A_ and D_2_ receptors can form heterodimers (71) with antagonistic A_2A_-D_2_ interactions within the receptor complex (72). Thus, it can be hypothesized that A_2A_ and D_2_ receptors could give rise to receptor complexes in astrocytes as well. The first demonstration of RRI between native A_2A_ and D_2_ receptors in astrocytes was recently provided by Cervetto and collaborators (73). In their study, A_2A_ and D_2_ receptors co-localized in the same striatal astrocytes, where they functionally interacted in the control of glutamate release. The results also suggested that this interaction involved the formation of A_2A_-D_2_ heterodimers, since administration of the synthetic peptide VLRRRRKRVN, which is able to interfere with the D_2_ receptor domain involved in electrostatic interactions critical to receptor heteromerization (74, 75), eliminated the A_2A_-mediated inhibition of the response to D_2_ receptor activation.

Further evidence of RRI between GPCRs in astroglial cells has emerged from studies on adenosine A_1_ and P2Y_1_ purinergic receptors (76, 77). These studies revealed a high level of co-localization and reciprocal functional interaction of the two receptors in human hippocampal astrocytes. Furthermore, co-immunoprecipitation data indicated the existence of A_1_-P2Y_1_ heteromeric complexes in the cells.

## GPCR Complexes in Peripheral Cells and Tissues

While GPCR complexes in the CNS have been the subject of considerable research, their identification and the characterization of their functional features in peripheral tissues have so far received less attention. There is, however, significant evidence that GPCR oligomerization could play a major role in the physiology and pathology of other districts of the organism. Available examples are summarized in Table 1.

**Table 1:** Examples of GPCR complexes in peripheral cells and tissues.

**Cell or tissue**	**Receptor complex**	**References**
Cardiomyocytes	AT_1_-β_2_	(78)
Renal mesangial cells	AT_1_-B_2_	(79)
Smooth muscle cells	AT_1_-P2Y_6_	(80)
Sympathetic neurons	AT_1_-α_2c_	(81)
Stellate hepatic cells	AT_1_-CB_1_	(82)
Gonads	LHR-LHR, FSHR-FSHR	(83–85)
	LHR-FSHR	
Pancreatic β islet cells	GHSR-SST_5A_	(86)
Carotid body	A_2B_-D_2_ (putative)	(87)
Cancer cells	GHSR-NTS_1_	(88)
	CB_2_-GPR_55_	(89)

In this respect, studies on the angiotensin II type 1 receptor (AT_1_) are of particular interest [see (90)]. AT_1_ has a central role in vascular homeostasis, since it supports the structural and functional integrity of the arterial wall; however, it is also implicated in the pathogenesis of hypertension (91, 92). AT_1_ has been reported to heterodimerize with various other GPCRs [see (90)], suggesting that a cross-regulation arises between angiotensin II and other signaling pathways. Heteromerization has been predicted to involve the fourth to seventh TM domains of the receptor (93), and the DRY ligand-binding motif of AT_1_ seems to be critical to the functional activation of signaling from oligomerized AT_1_ (94). Of relevance, in this context, was the indication of the existence of heterodimers between AT_1_ and β-adrenergic receptors in cardiomyocytes and related cell lines (78), where a single antagonist (AT_1_ or β-adrenergic receptor antagonist) proved able to induce a inhibition of both receptors. It has also been shown that the contribution of AT_1_ to specific forms of hypertension is modulated by the formation of receptor complexes with the B_2_ bradykinin receptor (79) in renal mesangial cells, and with purinergic P2Y_6_ receptors in mouse smooth-muscle cells (80), while physical interactions with the apelin receptor have been proposed to regulate the effect of angiotensin II in mouse models of atherosclerosis (95). A sure sign of major cardiovascular diseases that contribute to cardiac dysfunction is the hypersecretion of noradrenalin (NA). In this regard, the receptor complex between AT_1_ and the α_2C_ adrenergic receptor in sympathetic neurons was found to be involved in NA secretion, since the dual occupancy of the protomers by agonists produced a heterodimer conformation different from that induced when a single protomer was activated; this triggered atypical G_s_-cAMP-PKA signaling, promoting NA hypersecretion (81). Taken together, these findings suggest that receptor complexes involving AT_1_ may be promising targets for novel treatments of cardiovascular diseases (96) especially in hypertension and preeclampsia (97, 98).

Apart from its role in blood pressure regulation, AT_1_ contributes to the development of fibrosis in a number of organs (90). For instance, it is well-expressed in activated hepatic stellate cells, which are primary agents of the fibrogenic response in the liver (99). It has been shown that the AT_1_-mediated increase in profibrogenic markers in hepatic stellate cells of rats chronically treated with ethanol is completely blocked by an antagonist of the cannabinoid receptor CB_1_. These data have prompted the analysis of interactions between these two receptors, and the heteromerization of CB_1_ and AT_1_ receptors in this cell type has been demonstrated by means of co-localization, co-immunoprecipitation and BRET assays (82). Analysis of the signaling properties of the heteromer has shown that AT_1_ receptor agonists induce a rapid, dose-dependent increase in ERK1/2 phosphorylation, which is potentiated by CB_1_ receptor agonists and blocked by CB_1_ antagonists, suggesting that the CB_1_-AT_1_ heteromer may be a possible novel therapeutic target in the treatment of liver fibrosis.

Key players in the regulation of the cardiovascular system [see (100)] are endothelin and serotonin receptors. These are both expressed in many cardiovascular tissues, and *in vitro* results (mainly of a functional type or obtained on cell lines) have suggested that they could be part of receptor complexes (101, 102). In native cells and tissues, however, their involvement in heteromerization processes remains to be assessed.

Very recently, it has also been hypothesized (87) that receptor complexes exist in the carotid body (CB), a small peripheral chemoreceptor that plays a basic role in conditions such as hypercapnia, hypoxia, hypoglycemia and acidosis, in which it triggers an adequate cardiovascular and respiratory response. This hypothesis is based on the large repertoire of GPCRs expressed (most of which are able to form receptor complexes in other tissues) and on functional data providing indirect evidence of the existence of GPCR complexes in the CB. Specifically, an antagonistic RRI between dopamine D_2_ and adenosine A_2B_ receptors in CB type I cells has been suggested. Indeed, it has been shown that D2 agonists reduce catecholamine release and inhibit cAMP production in these cells, and that these effects are prevented by adenosine A_2B_ receptor agonists. Conversely, A_2B_ receptor antagonists counteract the increased catecholamine release induced by D_2_ antagonists (103, 104).

GPCRs are also of central importance in the endocrine system [see (100, 105)], and increasing evidence points to GPCR oligomerization as a significant aspect of endocrine regulation [see (106) for a recent detailed review]. For instance, a growing number of reports have suggested that GPCR heterodimerization may play significant roles in reproduction, including the secretion of hormones and the growth and maturation of follicles and oocytes [see (107) for a review specifically addressing this topic]. Indeed, several GPCRs are involved in the regulation of reproductive functions at the level of the reproductive organs and the hypothalamic-pituitary axes. Luteinizing hormone (LH), which is secreted by the adenohypophysis, stimulates testosterone production in Leydig cells of the male, and in females triggers ovulation by acting on the LH receptor (LHR), a class A GPCR. Biophysical and pharmacological assays have shown that LHR homomers displaying negative cooperativity between the receptor partners can be formed *in vitro* (83) and more recently a trans-complementation assay has been used to investigate the presence of LHR homomers and their functional relevance *in vivo* (108). To regulate pubertal maturation and reproductive processes, LH acts together with follicle-stimulating hormone (FSH); FSH is also produced by the anterior pituitary and binds the class A GPCR FSH receptor (FSHR). On the basis of crystallographic data, it has been hypothesized that FSHR has a dimeric structure and that, upon binding, it gives rise to a tetrameric complex composed of an FSH dimer that bridges the dimeric FSHR (109). Subsequent studies have pointed to a central role of the TM region of FSHR in stabilizing constitutive dimers (110). More recently, BRET assay (85) and fluorescence correlation spectroscopy (84) have also revealed heteromers between LHR and FSHR, in which heteromerization leads to an enhanced ligand dissociation rate and a negative regulation of cAMP production (84). LHR-FSHR receptor complexes are of potential physiological significance in females, since during the peri-ovulatory period co-expression of these receptors primarily occurs in granulosa cells [see (105)]. GPCR heteromers also impact on glucose metabolism, as indicated by FRET-based studies demonstrating heteromerization of growth hormone secretagogue receptor (GHSR) and somatostatin 5a receptor (SST_5a_) in β islet cells of the pancreas (86). In these studies, heteromerization changed the preferred G protein-coupling of GHSR from G_α*q*11_ to G_α*i*/0_, mediating the inhibition of the glucose-stimulated insulin secretion induced by ghrelin and somatostatin.

With regard to pathological tissues, the possibility of a GPCR heteromer-based strategy in oncology has been proposed by Moreno and collaborators (89). This is based on the finding that the cannabinoid CB_2_ receptor and the GPCR55 (GPR_55_) are overexpressed in cancer cells and human tumors and that they form heterodimers displaying antagonistic CB_2_-GPR_55_ interactions in cancer cells. Moreover, it has been shown that GHSR and neurotensin receptor 1 (NTS_1_) can establish direct structural interactions *in vitro*, and neuromedin-U has been indicated as a ligand for this heteromer (88). These findings are of interest to oncology. Indeed, in non-small cell lung cancer, it has been suggested that GHSR-NTS_1_ heteromers are involved in an autocrine growth-promoting pathway (88). Although preliminary, these data suggest that these heteroreceptor complexes may constitute novel targets in future cancer studies.

## Receptor Complexes Are Not Limited to GPCRs

**Figure 1:**
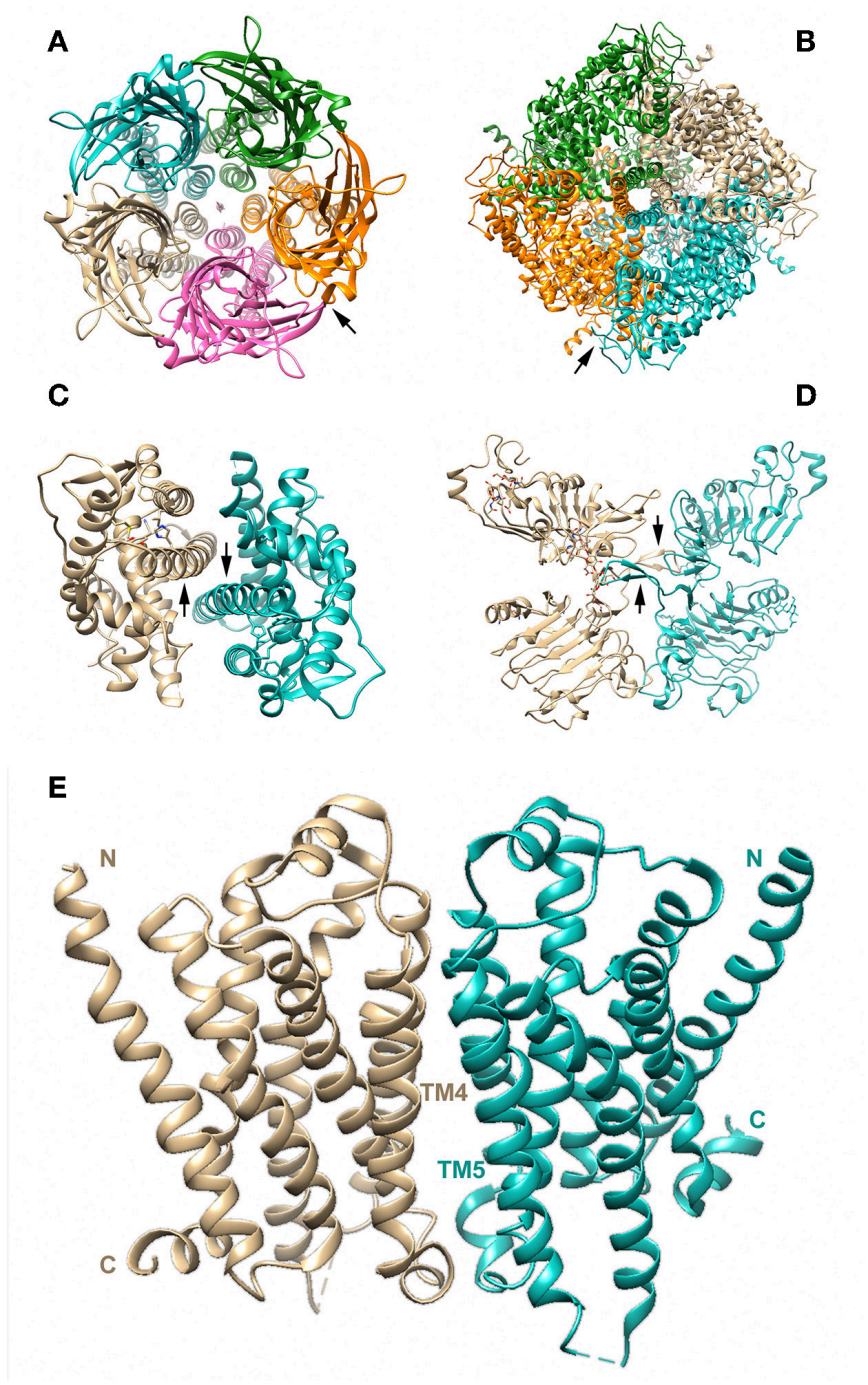
Multimeric molecular structures of receptors from different families, as determined by crystallographic studies. The protomers forming each complex are shown in different colors. **(A)** Top view (from the extracellular side) of a pentameric LGCI, namely a cationic ligand-gated ion channel [PDB code: 5HCJ; (112)]. The arrow indicates the interface between subunits, where the orthosteric binding site is located, halfway between the membrane and the top of the extracellular domain. **(B)** Bottom view of a tetrameric VGIC, the human transient receptor potential ion channel M_4_ [PDB code: 6BQV; (113)]. The arrow indicates the interface between neighboring monomers. The cytoplasmic domain involves four homology regions (MHR1 to MHR4) and MHR1 of one subunit interacts with MHR3 of the adjacent subunit to form the interface. **(C)** Dimeric HNR, the human estrogen receptor 1 [PDB code: 1X7E; (114)]. In each monomer, the arrow indicates helix 10/11, where the dimer interface is formed; **(D)** Dimeric extracellular domain of a human RTK, the EGFR [PDB code: 5WB7; (115)]. Arrows indicate the dimerization arms mediating dimer formation. **(E)** GPCR homodimer of β_1_-adrenergic receptors [PDB code: 4GPO; (116)]. N and C terminals are indicated. The dimerization interface has been shown to involve TM4 and TM5 (117). As illustrated, oligomerization plays an important role in the function of all receptor families, including GPCRs. Although GPCRs mostly signal as monomers, there might also be stable GPCR dimers/oligomers or transient quaternary structures that are constantly formed and dissociated at the cell membrane.

Advances in crystallographic techniques have revealed the structural architecture of many receptors. Although receptor proteins operating as monomers have been observed [see (111)] oligomeric organization appears to be quite a common feature in the different receptor families, as illustrated in Figure 1 [see (44) for a detailed review]. This probably constitutes an efficient mechanism for modulating the functionality of receptor proteins, including those able to signal as monomers, like GPCRs.

The LGIC family (see Figure 1A), for instance, mainly consists of constitutively pentameric ion channels (118), including nicotinic, serotonin and GABA_A_ receptors. Tetrameric and trimeric receptors are also part of this family (119). These include ionotropic glutamate receptors and purinergic P2X receptors, respectively. Although some homomeric LGICs exist, the majority of receptors in this family are hetero-oligomers made up of various subunits. The structures that have so far been characterized reveal strikingly similar 3D arrangements, showing features of symmetry with the ion channel lying along the central axis of symmetry (118) and ligand-binding sites mostly at subunit interfaces.

VGIC receptors also have an oligomeric structure [see (120)]. They are characterized by a α subunit (~260 kDa) that forms a large channel and one or two β subunits of 30–40 kDa. In addition to the well-known examples of VGIC, such as those for potassium, calcium, and sodium, the transient receptor potential (TRP) channels also belong to this family (121). These, however, are symmetrical homotetramers (Figure 1B) with a 3D structure resembling that of LGICs (122).

Regarding NHRs, these are ligand-regulated transcription factors with a disordered N-terminal domain, a central DNA-binding domain, and a C-terminal domain containing the pocket for the ligand. It is well-acknowledged that only one subset of NHRs is made up of monomeric receptors [see (123)], the majority of NHRs operating as homo- or hetero-dimers (Figure 1C).

Finally, RTKs (which function as receptors for growth factors and related hormones) all possess an extra-cellular domain of variable length that recognizes the ligand (Figure 1D), a single TM region and an intracellular domain linked to the tyrosine kinase domain, this latter performing the catalytic process which initiates signal transduction (124). With some exceptions, such as the insulin receptor (125), in the absence of a ligand most RTKs are monomeric; however, in almost all instances [some exceptions have been reported very recently, see (126)], dimerization is needed for their activation (127). Four mechanisms of dimerization have been hypothesized [see (44)]. These are: cross-linking of two receptor proteins by a bivalent ligand (e.g., nerve growth factor binding to its TrkA receptor); bivalent ligand binding combined with interaction between specific interfaces on the receptors to form the dimer (as when stem cell factor binds to the KIT receptor); the need for multiple contacts involving the agonist, the receptor and accessory proteins (e.g., FGF and its receptor); and “unmasking” of buried dimerization interfaces following the conformational rearrangement induced by ligand binding (e.g., EGF and its receptor). Due to this variety of possible mechanisms underlying RTK dimerization, it has been suggested that both symmetric and asymmetric arrangements of the extracellular domains may occur (128). Moreover, some data suggest that some RTKs (e.g., the PDGF_β_ receptor) could form high-order aggregates (129) and also directly interact with other RTKs (130), such as the EGF receptor (EGFR).

Thus, as recently pointed out by Changeux and Christopoulos (44), oligomerization plays an important role in the function of all receptor families, with the ion channel receptors (where multimerization is necessary) being located at one end of the spectrum and GPCRs (Figure 1E) at the other. Indeed, GPCRs may signal not only as monomers, but also as stable dimers/oligomers, or give rise to transient quaternary structures, which are constantly formed and dissociated at the cell membrane [see (8)].

In this context, RRI involving receptors from different families are also of interest. It is well-known that receptors can functionally interact, without coming into contact with each other, through mechanisms of transactivation or by sharing signaling pathways (131, 132). Recently, however, the formation (by direct RRI) of receptor complexes involving an RTK receptor, the fibroblast growth factor receptor 1, and GPCRs such as the serotonin 5-HT_1A_ receptor (133) or the muscarinic M_1_ receptor (134) has been associated with increased neurite densities in hippocampal cell cultures after agonist coactivation. In striatal glutamate synapses, a direct structural interaction between dopamine D_2_ and NMDA receptors that leads to inhibition of NMDA receptor signaling has been identified (135). Furthermore, recent data have prompted speculation that a possible direct interaction takes place between hyperpolarization-activated nucleotide-gated (HCN) cation channels and D_1_ dopamine receptors in the prefrontal cortex. Indeed, HCN and D_1_ receptors are co-localized in layer III of the dorsolateral prefrontal cortex and blocking the HCN channels has been seen to prevent the inhibition of neuronal firing induced by D_1_ signaling. Correspondingly, the blockade of HCN channels in the prefrontal cortex of rats has proved able to prevent working memory impairments induced by D_1_ stimulation or pharmacological stress (136).

## RRI as Allosteric Interactions

A clear discussion of allostery in receptors has recently been provided by Changeux and Christopoulos (44), and, for what concerns GPCR homomers and heteromers, extensive reviews have been provided by Kenakin and Miller (137) and by Smith and Milligan (138). Here, some basic concepts will be briefly summarized.

Allostery [see (139)] is a mode of communication between distant sites in proteins, in which the energy associated with dynamic or conformational changes at one site can be transported along specific pathways within the structure of the protein to other sites, which change their dynamic or conformational properties accordingly (140). In this respect, receptor molecules are undoubtedly “allosteric machines” (141), since their activation mechanism involves the recognition of an extracellular signal at the ligand-binding domain, and the changes induced are transmitted to the biologically active site of the protein, which, as in transmembrane receptors, may be located tens of Å away. Since changes in protein conformation underlie allosteric processes, the possibility for a protein to be allosterically modulated depends on its ability to acquire new conformations. Therefore, a protein with a rigid structure is less predisposed to be allosterically modulated than one that possesses segments that do not fold into a stable secondary structure, i.e., segments endowed with a high degree of intrinsic disorder (142, 143). Intrinsically disordered regions have been identified in all classes of membrane receptors. Mechanisms of structural change from order to disorder (or vice versa), for instance, have been hypothesized to underlie the activation of receptors of the RTK family (144) and intrinsic disorder of the N-terminal domain appears to play a significant role in the functionality of NHRs [see (145)]. GPCRs exhibit disordered segments extracellularly (in the N-terminus) and large disordered sequences in the cytosolic region, mainly in the intracellular loops—particularly ICL3—and in the C-terminal domain (142, 146).

Malleability and structural plasticity, however, are of importance not only because they enable conformational fluctuations and intra-receptor interactions to take place, but also because they allow the formation and dynamics of receptor complexes. Indeed, when two protomers establish direct RRI, thereby giving rise to a quaternary structure, the energy associated with a perturbation at one site of one protomer can propagate over the interface between receptors into the nearby protomers, thus changing their conformation and functional features and leading to a cooperative behavior of the complex (147).

Identifying the residues that specifically interact to form the interaction interface is therefore of significant interest in current research on receptor oligomerization (148) as these residues influence the models of potential allosteric interactions between receptor partners.

## Interaction Interfaces

Pentameric LGCIs derive from the assembly of subunits containing an N-terminal extracellular domain (ECD), four transmembrane segments (named M_1_ to M_4_) and a cytoplasmic domain between M_3_ and M_4_ of highly variable sequence and length (118). To ensure the correct assembly of the channel, a very specific inter-subunit interface is formed in the extracellular domain through mixtures of salt bridges, van der Waals contacts and hydrogen bonds (149). In the GABA_A_ receptor, for instance, inter-subunit contacts between the central portion of the ECD involve β_4_, β_5_, β_5_′, and β_6_ strands and flanking loops (149). The same concept can be applied to trimeric (150) and tetrameric (151) LGCIs. AMPA-type glutamate receptors are an example (151). Subunits first form dimers, which subsequently assemble into tetramers. Dimerization is driven by specific interfaces in the most superficial layer of the extra-cellular region (the N-terminal domain), while tetramerization is mediated by contact points in all layers of that region. By contrast, specific interfaces in the cytoplasmic region of the receptor complex are implicated in the assembly of VGCIs (152, 153). Studies of the TRPV_6_ channel, for instance, have identified a domain encompassing an ankyrin repeat in the intracellular region of the monomers; this domain is key to mediating the correct assembly of the subunits in order to obtain a functional channel (153).

The superfamily of nuclear receptors is composed of ligand-dependent transcription factors. These regulate a diversity of cellular processes, including development, differentiation, growth, metabolism, and reproduction. Nuclear receptors are proteins composed of a C-terminal ligand-binding domain (LBD), a conserved DNA-binding domain (DBD), and a variable amino-terminal region (154). They operate as homo- or hetero-dimers, binding to hormone response elements of target genes. A specific dimerization interface (also named D box) resides within the DBD and corresponds to a zinc-binding module (155).

As mentioned earlier, RTKs are single-pass trans-membrane proteins with an extracellular N-terminal domain containing motifs involved in ligand binding. The TM domain is followed by a juxta-membrane region and an intracellular catalytic domain. RTKs operate as dimers, and helix-helix interactions in the TM domain are key to providing the stability of full-length dimers and maintaining a signaling-competent dimeric conformation (156, 157). Specifically, as observed in the FGF_3_ receptor (158) and the ErbB_2_ EGFR (156), *GxxxG* motifs, also called *SmallxxxSmall* motifs, are part of the dimer interface. These motifs are characterized by the presence of small amino acids (Ala, Gly, Ser, and Thr) in *i, i*+*4* positions and drive interactions between hydrophobic helices in membranes (157).

In comparison with the other receptor families, GPCRs are endowed with some distinctive features in terms of interfaces for dimerization. Our knowledge of interaction interfaces has been extended both through the application of bioinformatics methods [see (8, 159)], in order to predict amino acid sequences potentially involved, and by experimental investigation. Indeed, recent improvements in experimental procedures have provided researchers with a range of methods and tools for identifying and characterizing interaction interfaces in GPCRs. Significant advances in GPCR crystallization techniques, for instance, have led to an increase in the number of experimentally assessed structures in recent years (160). Further experimental tools that are currently available include: atomic force microscopy (147); new super-resolution imaging approaches, such as photoactivated localization microscopy (PALM) (161); far-UV CD spectroscopy, and SDS-PAGE using synthetic peptides corresponding to different transmembrane domains (162). By using mass spectrometry combined with collision-induced dissociation experiments, Woods et al. (74, 75) investigated intracellular domains (e.g., ICL3 and C-terminus) and demonstrated strong electrostatic interactions in GPCR heteroreceptor complexes. Experimental results concerning dimerization interfaces are reported in Table 2 for a number of GPCRs.

**Table 2:** Examples of experimentally assessed dimerization interfaces in GPCRs.

**Receptor**	**Domains involved**	**References**
Adenosine A_1_	TM4, TM5, TM6	(163)
Adenosine A_2A_	TM4, TM5, TM6	(164)
	ICL3, C-terminus	(68)
Adrenergic β_1_	TM1, TM4, TM5	(116)
		(117)
Adrenergic β_2_	TM1, H8	(36)
Cannabinoid receptor 1	TM4, TM5	(165)
	ICL3	
Chemokine receptor 5	TM1, TM4	(166)
Dopamine D_2_	TM1, TM4, TM5	(167)
	ICL3, C-terminus	(68)
δ-opioid	TM4, TM5	(168)
μ-opioid	TM1, TM2, TM5, TM6	(37)
Muscarinic M_3_	ICL3	(169)

*H8, C-terminal amphipathic helix 8*.

The first noteworthy feature to emerge from both computational and experimental studies concerns the ability of GPCR structures to interact via multiple interfaces. The A_2A_-D_2_ heteromer is probably an example of this. In the study by Woods and coworkers (74) dimer formation was found to occur at the intracellular level through electrostatic interactions between the ICL3 of D_2_ and the C-tail from A_2A_. Very recently, however, the interaction between TM4 and TM5 helices was also shown to support the heteromerization of these receptors (164). In quite a large number of GPCR complexes, TM4, TM5, TM6, and ICL3 were found to be the main interfaces. Regarding the possible involvement of extracellular loops in RRI, this has been demonstrated for some class A GPCRs (116), while in some class C GPCRs, interactions between extracellular domains through disulfide bridges (29) have been demonstrated.

A further interesting finding to emerge from computational and experimental studies on GPCRs oligomerization is the presence at the interface of motifs that appear to be of particular importance in the allosteric interaction. As demonstrated by Woods et al. (75, 170, 171), electrostatic interactions between intracellular domains may occur between a positively charged arginine (Arg)-rich motif of one receptor and a negatively charged serine-phosphate-containing motif of another receptor. Once established, this interaction possesses a covalent-like stability, which probably constitutes a significant mechanism for the assembly of the receptor complex. As in RTKs, *Small-xxx-Small* motifs have been reported to promote TM1 self-association in some GPCRs (172), and, by means of a bioinformatics approach, Tarakanov and Fuxe (173) identified a set of triplet homologies, mainly located at the receptor-receptor interface, that may be responsible for RRI. Most of these are motifs containing leucine. Another set of triplets contains charged amino acids. It has been suggested that the electrostatic interaction between triplets may guide and clasp the interactions between protein partners (51, 174).

The evidence that a given GPCR can exploit multiple interaction interfaces implies at least two significant consequences with regard to the architecture of the resulting receptor complexes:

- The first concerns the number of subunits forming the complex (i.e., its stoichiometry), since the possibility exists that a given GPCR could be involved in oligomeric assemblies of different orders (48). Among the first to provide evidence of the role played by interaction interfaces between protomers in arranging the quaternary structure of receptor complexes were Navarro and coworkers (165). Their study focused on dopamine D_2_, adenosine A_2A_, and cannabinoid CB_1_ receptors. Each of these possesses two intracellular domains that are able to specifically interact with intracellular domains of the other two protomers via electrostatic interactions, leading not only to the formation of dimers (A_2A_-D_2_, A_2A_-CB_1_, CB_1_-D_2_) but also to the assembly of an A_2A_-D_2_-CB_1_ heterotrimer. Indeed, trimeric receptor complexes have been identified (175); examples include the muscarinic M_2_ homotrimer (176), the A_2A_-D_2_-mGlu_5_ (177) heteroreceptor complex, the dynamic Gal_1_-5HT_1A_-GPR_39_ heterotrimer (178), and the putative Gal_1_-Gal_2_-5HT_1A_ heterotrimer (179). With regard to tetrameric arrangements, the possible occurrence of a heterotetrameric structure for the complexes formed by adenosine A_1_ and A_2A_ receptors has recently been proposed (163). In this complex, homodimerization is supported by a TM4-TM5 interface, and a TM5-TM6 interface mediates heterodimerization. Evidence that tetrameric assemblies of β_2_-adrenergic receptors (β_2A_R) occur spontaneously following reconstitution into phospholipid vesicles (36) was provided by Kobilka et al. who suggested that oligomerization was an intrinsic property of β_2A_R. Evidence of higher-order GPCR oligomers has also been reported. Combined BRET/FRET and complementation studies, for instance, have revealed that, in the plasma membrane of living mammalian cells, the association of dopamine D_2_ receptors by means of symmetrical interfaces at TM4 and TM1 can generate an assembly composed of at least four protomers (167). Moreover, studies based on the analysis of PALM data have led to the hypothesis that, depending on the specific membrane microenvironment, direct RRI among GPCRs could allow the formation of high-order oligomers, such as tetramers, octamers, and complexes of larger size (180).
- Secondly, the notion that GPCRs can exploit multiple interaction interfaces opens up the possibility that a given set of interacting GPCRs could associate according to different geometrical arrangements (181); these associations would depend on a variety of conditions that include not only the physical features of the protomers involved (hydrophobicity, surface charge, etc.) but also the characteristics of the microenvironment surrounding the interacting monomers. The functional behavior of a receptor complex may be significantly influenced by its topological arrangement. In this regard, Agnati et al. carried out a theoretical analysis based on thermodynamic considerations and which focused on the role that the spatial arrangement of GPCR monomers may play within a receptor complex (182). They showed that, for each given set of binding and interaction constants, the theoretical saturation curves of trimeric or tetrameric receptor complexes were dependent on the geometry of the assembly formed. Interesting experimental evidence of this concept was recently provided by Jonas et al. (183), who adopted a super-resolution imaging approach. Their study focused on two mutant luteinizing hormone receptors that can function only via intermolecular cooperation in which the oligomeric forms are favored over the dimeric ones. Their PD-PALM images of trimers and tetramers showed that monomers associated through helix interfaces according to a variety of distinct spatial arrangements that were also different from one another in terms of signal sensitivity and strength.

## Pharmacological Features of the Receptor Complexes

The importance of supramolecular assemblies of receptors can be appreciated when we consider the possible emergence of integrative functions from the collective dynamics of a receptor complex (147). Indeed, a configuration change in a given protomer due to allosteric RRI will modulate the probability of configuration change in the adjacent receptors in the complex, and propagation of this effect throughout the cluster will lead to an integrated regulation of multiple effectors (184). These concepts have been well-described by mathematical models of cooperative dynamics in receptor assemblies [see (8, 159) for reviews], based on discrete dynamics (49) or on thermodynamics-based approaches (185). These models have allowed receptor complexes to be described as possessing “emergent properties”, i.e., biochemical and functional features that could not be fully anticipated on the basis of the characteristics of the single receptor partners. According to a metaphor proposed by Kenakin (186), since receptor complexes are not just “on-off” switches but exhibit quite a high ability to elaborate incoming information, they would operate as a sort of molecular “microprocessor”.

**Figure 2:**
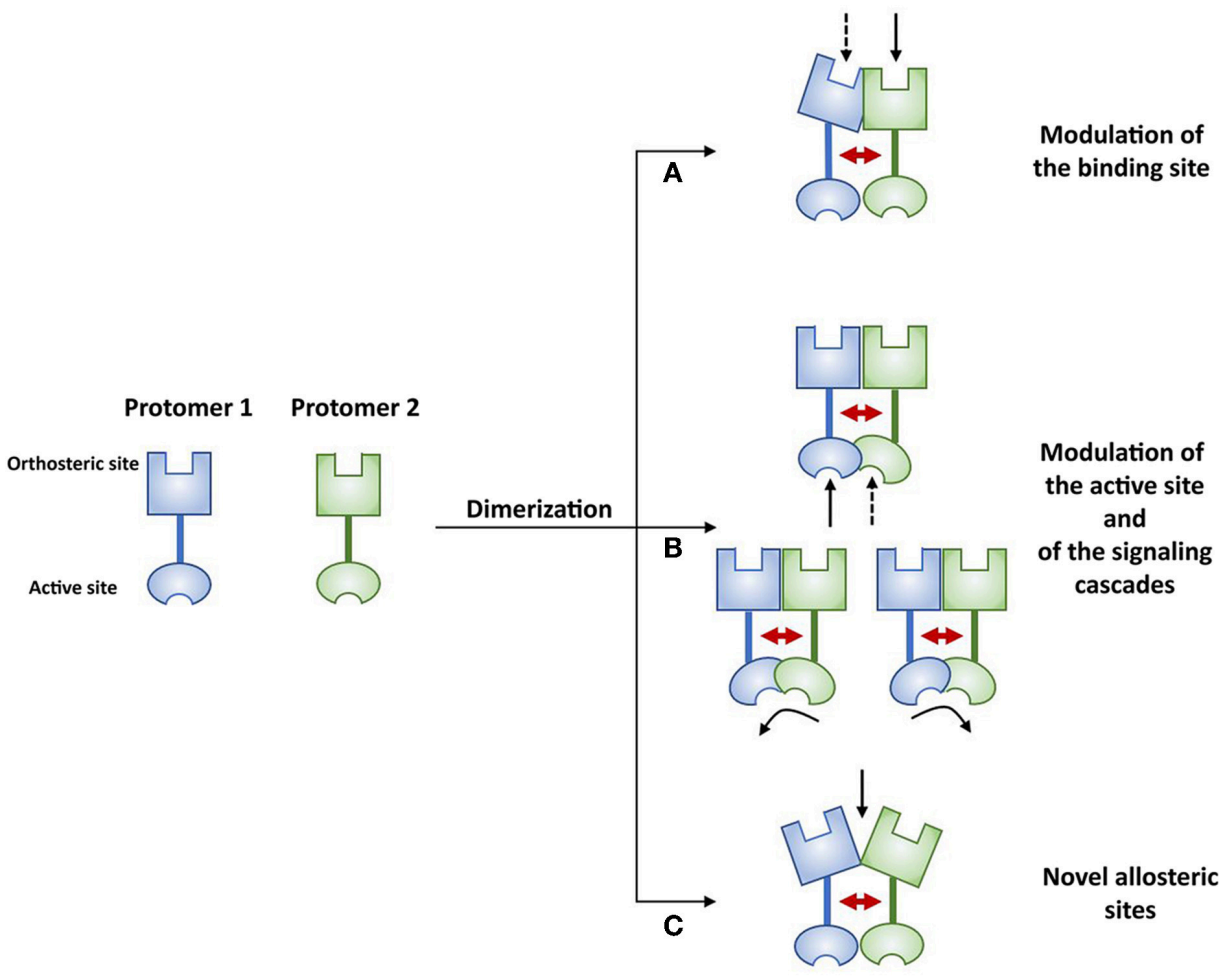
As a result of allosteric RRI, receptor complexes appear to be endowed with pharmacological features that cannot be fully derived from the characteristics of the single participating protomers (see text).

Thus, when RRI take place at the membrane, the actual signaling outcomes of receptor complexes depend on several factors, including the composition of the complex and its topological organization, the traffic of the receptor complex, the effects of ligands on the formation of the assembly and on its stability, and, quite often, crosstalk with alternative signaling pathways (48, 187). Together, these factors may strongly influence the chain of events linking ligand recognition to signal transduction from the single protomers. Figure 2 schematically summarizes some of the potential signaling consequences of the allosteric modulations occurring when a receptor complex forms.

**Figure 3:**
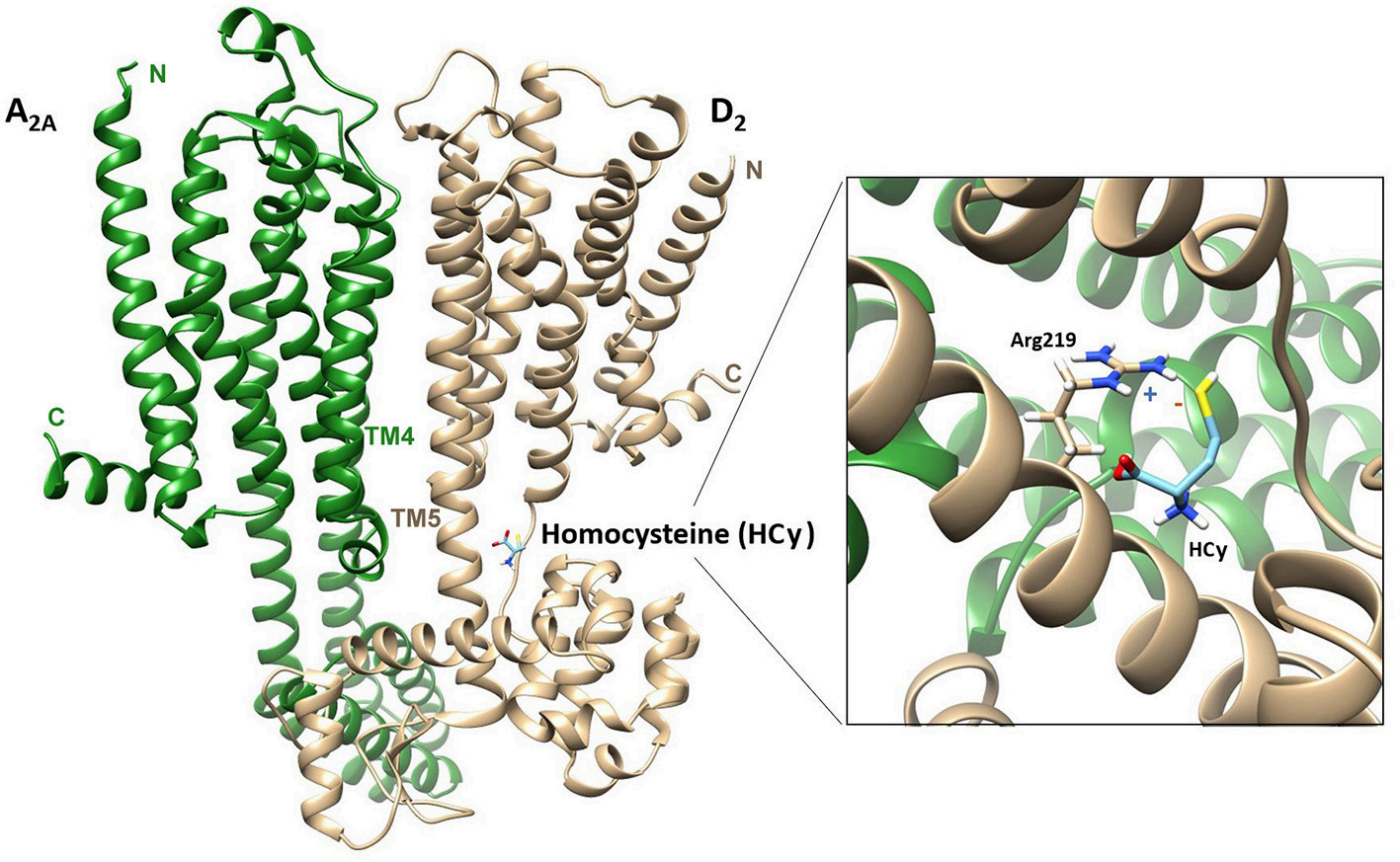
Docking [obtained by means of the Rosetta software, (203)] of the crystallographically-assessed structures of adenosine A_2A_ [PDB code: 4EIY; (204)] and dopamine D_2_ [PDB code: 6CM4; (205)] arranged to form the A_2A_-D_2_ heterodimer through a TM4-TM5 interface as recently described by (164). The docking of homocysteine (HCy) to the receptor complex is also shown. This occurs in an Arg-rich region [the epitope _115_VLRRRRKRVN] of the D_2_ receptor ICL3 and is consistent with an electrostatic interaction between the negatively charged thiol group in HCy and the positively charged guanidinium group of arginine (201).

These can be briefly summarized as follows [see (187) and, with regard to GPCRs, (7, 8, 28, 53) for reviews]:

- In a variety of receptor complexes, modulation of the binding sites has been reported as a consequence of allosteric RRI. One of the first examples was the A_2A_-D_2_ heterodimer, where the binding of the adenosine A_2A_ agonist CGS21680 reduced the affinity of the dopamine D_2_ agonist-binding site (188). In this GPCR heterodimer, the interaction between D_2_ and A_2A_ is reciprocal, since the A_2A_-induced increase in cAMP accumulation via G_i/o_ at the level of the adenylate cyclase is inhibited by D_2_ receptor activation (189). A similar reciprocal modulation occurs in the CCR_2b_-CCR_5_ chemokine receptor dimer. When this heteroreceptor complex forms, the CCR_5_, which is normally insensitive to monocyte chemoattractant protein-1 (MCP-1), becomes able to bind MCP-1. Likewise, the CCR_2b_ receptor, which is normally unresponsive to the CCR_5_ chemokine ligand macrophage inflammatory protein-1β (CCL4), binds CCL4 when in complex with CCR_5_ (190). Modulation of the binding sites consequent to subunit assembly may also occur in RTKs, as suggested by studies (191) on the insulin receptor (IR). The human IR is a glycoprotein that exists as two isoforms, which have a dimeric structure consisting of two α subunits and two β subunits linked by disulfide bonds. It is transcribed from a single gene encoding both α and β subunits. The two IR isoforms differ by 12 amino acids, which are absent (IR-A) or present (IR-B) at the C-terminal part of the α subunit. IR-A and IR-B exhibit at most a 2-fold difference in insulin affinity, but the two hormones, insulin-like growth factor 1 and insulin-like growth factor 2, have been found to have up to 5-fold higher affinity for IR-A than for IR-B.
- Changes in the decoding of signals reaching protomers constitute a second mechanism induced by allosteric RRI. This aspect seems to be of particular importance in GPCRs. Indeed, many functional/pharmacological and structural-based studies have shown that a GPCR does not act as a simple switch that turns a given signaling pathway “on” or “off”; rather, it can assume multiple conformations once it is bound by a given ligand or through interactions with other signaling partners. This means that GPCRs are multidimensional transducers that can engage, and differentially regulate, diverse signaling pathways, such as distinct G protein classes or β-arrestins. The discovery of molecules able to activate distinct pathways after interacting with the same receptor led to the concept of functional selectivity and biased agonism, which was used to describe these GPCR-based signaling processes [this topic was recently extensively reviewed by Costa-Neto et al. (192), Pupo et al. (193), Goupil et al. (14)]. Thus, when a receptor complex forms, the pattern of possible configurations that each GPCR protomer can assume is influenced not only by the ligands, but also by RRI with the other partners in the complex, potentially leading to functional selectivity of signaling downstream (14, 137). Changes in the decoding of signals associated to GPCR complex formation have been reported. The heterodimer formed by dopamine D_1_ and histamine H_3_ receptors provides a first example (194). In the experimental conditions used in this study, when the receptor complex forms, the D_1_ receptor changes its coupling from the G_s_ to the G_i_ protein, to which H_3_ receptors are already coupled. As a consequence, in the presence of the H_3_ receptor, D_1_ receptors can no longer activate adenylyl cyclase, but, being coupled to G_i_, they transduce the signal toward the MAPK pathway. The recruitment of G proteins other than those expected for the monomers has been observed after D_1_-D_2_ dimerization (195) and a switch between G protein and β-arrestin signaling (196) has been documented after κ-μ and κ-δ opioid receptor heteromerization (197). Processes of this type can also be hypothesized in some RTKs. IR and the closely related insulin-like growth factor receptor 1 (IGF_1_) are present in the membrane as preformed dimeric complexes, and both bind insulin and members of the insulin-like peptide family. Signaling through IR and IGF_1_, however, has different physiological outcomes [see (187)], with IGF_1_ signaling being essentially mitogenic (through the Ras/MAPK pathway) and IR signaling mainly producing metabolic effects (through the PDK/Akt pathway). The EGFR provides a further example. Crystallography and other approaches (115) have shown that different ligands stabilize different dimeric conformations of the EGFR extracellular region, leading to different signaling dynamics.
- A relevant aspect of receptor complex formation is the possibility that novel specific allosteric sites suitable for the binding of some modulators could appear in the quaternary structure resulting from the assemblage of the protomers. Thus, ligands specific to the receptor complex as such may also exist [see (96)]. Since the early discovery of benzodiazepines as allosteric activators of the GABA_A_ receptor, it has been shown that, in addition to the orthosteric site, most constitutively dimerized/oligomerized cellular receptors possess spatially distinct sites that modulate their allosteric transitions. Pharmacologically, allosteric ligands can be classified as “positive allosteric modulators” (PAM), when they enhance the effect of the orthosteric ligand, “negative allosteric modulators”, when they reduce the effect of the orthosteric ligand, and “neutral allosteric ligands”, if their binding to the allosteric site does not modulate the effect of the orthosteric ligand. Sometimes a PAM may activate the receptor even in the absence of an agonist, and is therefore referred to as an “allosteric agonist”. Combinations of these properties are also possible [see (44) for a discussion of the topic]. The same concepts apply to GPCR monomers, where allosteric binding sites may be present in various domains of the protein (198). Allosteric binding sites of class A GPCRs are, in most cases, located in the same region as the orthosteric site (i.e., within the seven-transmembrane domain), while the two types of sites are usually well-separated in class C GPCRs [see (199)]. The formation of a GPCR receptor complex, however, can result in significant structural and functional changes in the allosteric binding sites on single monomers [see (200)] and in the appearance of new allosteric sites. In this respect, a first example of the possible existence of allosteric modulators specific to a GPCR receptor complex was provided by studies on the effect of homocysteine (142, 201, 202) on the A_2A_-D_2_ heterodimer (Figure 3). In Chinese hamster ovary cells stably cotransfected with dopamine D_2_ and adenosine A_2A_ receptors (201) homocysteine was found to selectively reduce the internalization of the receptor complexes induced by D_2_ receptor stimulation, and in astrocytes (202) homocysteine reduced D_2_-mediated inhibition of glutamate release without altering the A_2A_-D_2_ interaction, since the A_2A_-mediated antagonism of the D_2_ effect was maintained. Mass spectrometric analysis (201) provided mechanistic support for these findings. This revealed that, by exploiting an Arg-thiol electrostatic interaction, homocysteine formed non-covalent complexes with the two Arg-rich epitopes of the ICL3 in the D_2_ receptor, one of which was also involved in the dimerization interface. FRET experiments, however, showed that homocysteine was unable to disrupt or prevent the heteromerization of A_2A_ and D_2_ receptors, suggesting that it probably behaves as a modulator of the allosteric process of energy transmission between the two partners.

A final aspect that deserves to be mentioned [see (8, 187) for a more detailed discussion] is the cell environment in which receptors and receptor complexes are located. Indeed, their signaling outcome is also influenced by the network of molecular interactions they can establish with other biochemical components. For what concerns membrane receptors, the term “horizontal molecular network” (48) has also been proposed to illustrate this concept. By 2003, 50, or more GPCR interacting proteins (GIP) had already been discovered and, in a review article, Bockaert et al. (206) drew attention to the C terminal tail of the GPCRs as an important site for the establishment of functional protein networks. The available findings indicate that receptor complexes are often involved in multiple receptor-protein interactions that may influence their assemblage and stoichiometry [see (8)]. Many GPCR interacting proteins act as scaffolding or adapter proteins, modulating the physical receptor-receptor interactions in receptor complexes (207). An association of particular interest occurs between GPCRs and a set of three homologous transmembrane proteins, which have been named RAMP (receptor activity-modifying membrane protein) (208). When RAMPs associate with the calcitonin-like receptor (CLR), complexes with very different functional profiles are generated: the RAMP1-CLR complex behaves phenotypically as a calcitonin gene-related peptide receptor, whereas the assembly of RAMP2 or RAMP3 with CLR provides specificity for adrenomedullin (209). RAMPs have also been shown to associate with other B family GPCRs, including glucagon receptors and parathyroid hormone [see (137)]. With regard to nuclear receptors, within the cytoplasm they are often found to be complexed with other proteins, which act as co-activators or co-repressors, while within the nucleus, nuclear receptors are part of larger transcriptional regulatory complexes (210).

Thus, in view of the multiplicity of support proteins with which receptors operate within the cell, it is realistic to surmise that these support proteins could have a significant impact on the properties of the receptors.

For what concerns membrane receptors, the lipid environment is also important, since this has been shown to influence receptor function [see (8)]. For instance, several aging-related health disorders have been found to be associated to membrane composition changes that can alter GPCR signaling (211). Furthermore, membrane features may regulate receptor assembly in membrane nanodomains through hydrophobic interactions (212).

## Concluding Remarks

Intercellular communication is a key process in the physiology of living beings, and the fundamental mode of communication in biological systems involves interaction between specific receptors expressed by the target cells and chemicals or energy forms released by a source. Thus, it is not surprising that the majority of the drugs currently used to treat pathological conditions are basically agonists or antagonists of some classes of receptors. Until relatively recently, drug design was based on the concept that ligands compete for interaction with a common “rigid” site [see (213)]. The discovery of flexible allosteric proteins and of allosteric modulatory sites in all receptor families [see (44)] paved the way to the design of new drugs that interacted with topographically distinct active sites on the receptor protein, and which often provided greater selectivity in receptor targeting. Subsequently, GPCRs (the largest family of receptors) were found to be even more versatile allosteric machines than previously believed, being able to alter their configuration to accommodate ligands and engage distinct signaling effector subsets [see (192)]. Moreover, GPCRs were seen to operate not only as monomers, but also as quaternary structures (17, 19) in which the configuration of the single receptors and of the entire complex is shaped by networks of electrostatic interactions (hydrogen bonds, van der Waals forces), thereby enabling incoming signals to be integrated already at the plasma membrane level. Once established, these integrative mechanisms can change the function of the GPCRs involved, leading to a sophisticated dynamic of the receptor assembly in terms of modulation of recognition and signaling [see (28)]. However, further research is needed in order to gain a deeper understanding of the signaling features of GPCR complexes, in terms of their possible configurations and downstream signaling pathways, a goal which would undoubtedly be of substantial interest.

Although RRI have so far been mainly studied and characterized in central neurons, they appear to be a widespread phenomenon, contributing to the metabolic regulation of several cell types and tissues other than the CNS. Moreover, oligomerization is not limited to GPCRs, as demonstrated in the other receptor families, in which the active form of most of the receptors is the result of the proper dimeric/oligomeric association of protein subunits. Both of these issues warrant further research.

In addition [see (187)], increasing evidence has shown that responses to specific ligands are critically influenced by the environment in which receptors and receptor complexes are located, and, in particular, by other proteins and biochemical constituents that establish structural or functional interactions with them. Within this context, signaling cannot be viewed exclusively as the output of a single receptor-agonist pair; rather, it often results from the modification of the targeted receptor or receptor complex by scaffolding proteins and other signaling partners.

Taken together, these findings have at least two important consequences for the study of new pharmacological tools, in particular for what concerns GPCRs, which constitute the target of about 50% of currently available drugs (28). On the one hand, RRI may be potential sources of undesired side effects of new drugs that are assumed to be specific agonists or antagonists of a given receptor, since the fine-tuned integrated response obtained through allosteric RRI could lead to unexpected outcomes. Indeed, as pointed out by Kleinau et al. (106), future studies should strive to characterize the receptor complexes typically expressed in pathological human tissues and to carefully distinguish the functional effects induced by monomers from those induced by receptor complexes. On the other hand, however, RRI may provide new opportunities to optimize pharmacological treatments in terms of receptor targets and tissue selectivity or to develop completely new pharmacological interventions that specifically target receptor complexes. In this regard, very promising results have emerged from studies on high-affinity antibodies (214), ligands for allosteric sites unique to oligomeric assemblies (215), and bivalent ligands selective for dimeric receptor complexes (105, 216).

## Author Contributions

DG and LA projected the paper and DG wrote the text. DG, MM, CT, and GM performed bibliographic search and collected relevant sources. All the authors discussed and revised the text before submission.

### Conflict of Interest Statement

The authors declare that the research was conducted in the absence of any commercial or financial relationships that could be construed as a potential conflict of interest.
